# Structured Online Systematic Review Training for Medical Students: An Elective Module in Physiology

**DOI:** 10.7759/cureus.66078

**Published:** 2024-08-03

**Authors:** Arunima Chaudhuri, Asitava Deb Roy, Shivasakthy Manivasakan, Himel Mondal

**Affiliations:** 1 Institute of Health Professions Education, Sri Balaji Vidyapeeth, Puducherry, IND; 2 Department of Physiology, Burdwan Medical College and Hospital, Bardhaman, IND; 3 Department of Pathology, Mata Gujri Memorial Medical College, Kishanganj, IND; 4 Department of Physiology, All India Institute of Medical Sciences, Deoghar, Deoghar, IND

**Keywords:** competency-based medical education, national medical commission (nmc), mbbs student, training module, research writing, medical student, undergraduate student research, online module, systematic review, electives

## Abstract

Background

Recently, elective posting has been introduced by the National Medical Commission (NMC) of India in the undergraduate competency-based medical education (CBME) curriculum. To successfully implement it in medical colleges, facilitators (medical teachers) need to commit extra time. Hence, this study aimed to evaluate the impact of online teaching-learning methods for implementing an elective module for undergraduate medical students at Burdwan Medical College and Hospital, West Bengal, India.

Methods

An online module for systematic review methods was developed using the Delphi method. This module was used to train 30 medical students divided into six groups. One resident and one faculty facilitated each group. After the elective program of 15 days, program feedback and evaluation using the Kirkpatrick model were obtained from the students.

Results

A total of 30 undergraduate medical students with a mean age of 22.7±0.95 years participated in the study. All of them successfully conducted a systematic review per group. The students' feedback was 86.33% positive, and the project evaluation showed an 84% positive opinion. The highest score was for understanding, facilitators' knowledge, and experience. The lowest score was for the immediate applicability of the knowledge.

Conclusion

An online systematic review training module can be used for elective teaching-learning for final-year medical students, particularly within limited time and resource constraints. Students appreciated the module's clear objectives, appropriate complexity, and facilitators' expertise, leading to improved communication, engagement, and critical-thinking skills. Despite some limitations, these findings suggest that online learning can complement traditional methods and address logistical challenges in medical education, warranting further research on its long-term impact and broader applicability.

## Introduction

Electives are a vital component of undergraduate medical education, offering numerous benefits that contribute to the development of competent, compassionate, and well-rounded physicians [1]. They enhance the educational experience by providing opportunities for exploration, skill development, personalized learning, and professional growth. Recently, electives have been introduced by the National Medical Commission in the MBBS undergraduate competency-based medical education curriculum [2].

By experiencing different specialties, students can make more informed decisions about their future careers. Electives provide opportunities to develop a wide range of skills, from clinical techniques to research methodologies. Students can tailor their education to their interests and career goals, making their medical training more relevant and engaging [3,4].

Research experience helps students appreciate the importance of evidence-based medicine, which is crucial for making informed clinical decisions. Conducting research can lead to publications in medical journals and presentations at conferences, which are valuable for academic and professional growth. It provides opportunities to collaborate with experienced researchers and clinicians, fostering professional relationships [5-7]. However, medical students often face significant challenges in managing their time, especially when it comes to balancing electives with regular coursework [8,9]. In our institution, these students are allotted only 15 days for electives, from 2:00 to 4:00 p.m., while still attending their regular classes. This limited timeframe puts immense pressure on students, making it difficult to fully engage in elective modules that are crucial for their professional development. Additionally, the faculty members' availability is constrained during this period, as many are occupied with first-year students' practical classes. This scarcity of faculty resources further complicates the scheduling and delivery of elective sessions [10]. Given these constraints, conducting teaching and learning activities online presents a viable solution that benefits both faculty and students.

In this context, we designed a structured online systematic review training module for final-year medical students during their elective period in the Department of Physiology. The main objective was to evaluate the impact of online teaching-learning methods for implementing an elective module for undergraduate medical students.

## Materials and methods

Study design and participants

**Figure 1:**
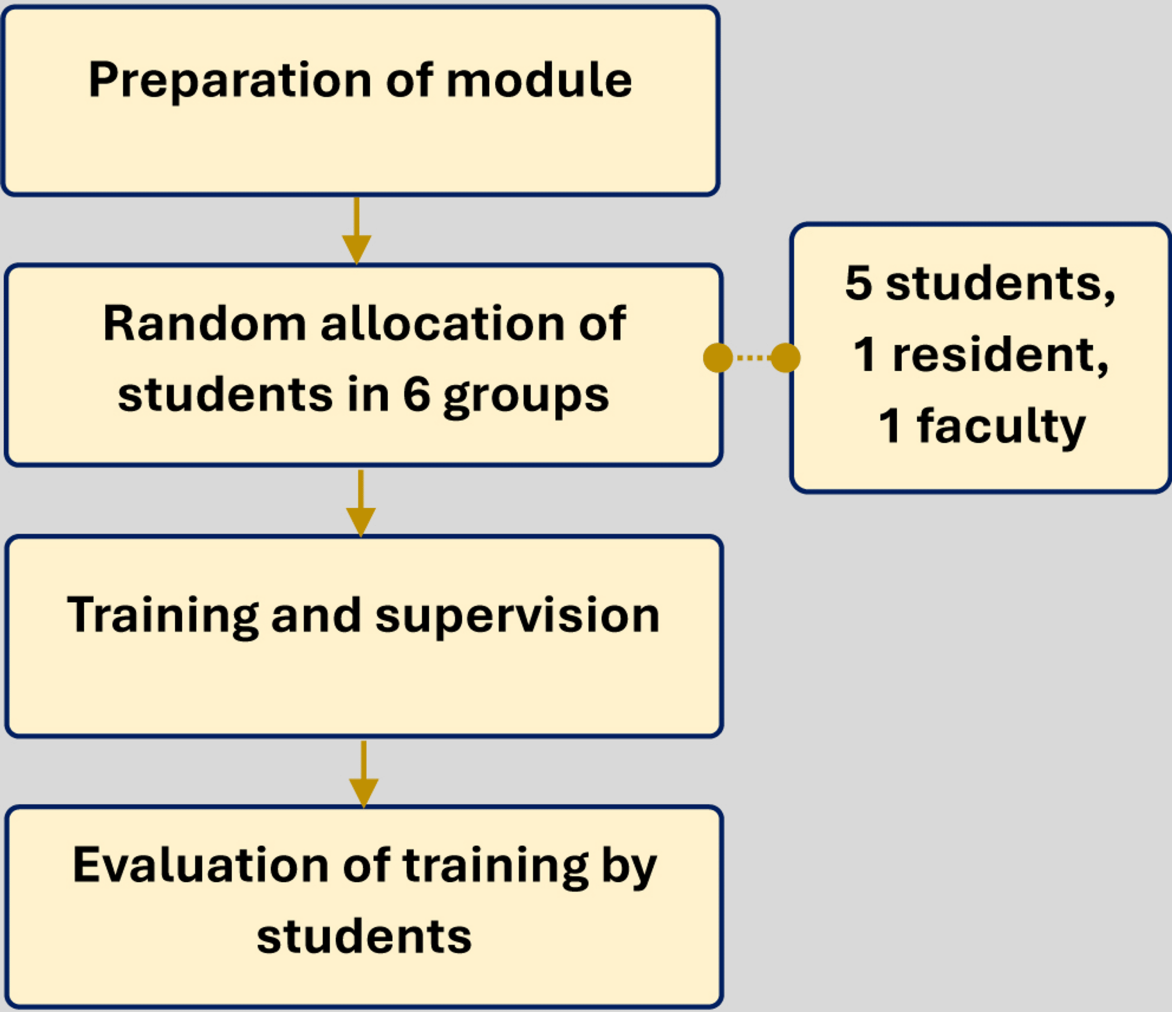
Brief study process

This study was conducted on 30 MBBS students from the 2021-2022 batch, posted in the Department of Physiology, Burdwan Medical College and Hospital, West Bengal, India, during their elective sessions of one month. Institutional ethical clearance was obtained (BMC/IEC/159 dated June 3, 2024), and informed consent was secured from all participants. Students were assigned to the Department of Physiology based on their roll numbers, as per the college authorities' decision. A total of 15 students were allotted to the department for 15 days, and in the next 15 days, another 15 students were allotted. The overall study procedure is shown in Figure 1.

Module development and topic selection

A systematic review module was developed by five faculty members using Delphi rounds and validated two months before the commencement of the study. Experts in medical education reviewed the module to ensure its relevance and rigor. The topics for systematic reviews were selected by senior faculty members, with significant input from students. The chosen topics were as follows: leadership styles and skills among undergraduate medical students, professionalism and ethics in medical education, academic leadership in medical education, use of social media in medical education, virtual reality and augmented reality in medical education, and ethical perspectives of the use of artificial intelligence in medical education.

Group formation and leadership

Students were randomly divided into six groups (three groups for 15 days and three groups for the next 15 days), each led by a resident (i.e., a postgraduate (PG) student). Faculty members continuously supervised group activities to ensure proper guidance and adherence to the module. Three faculty members from the institution and three from external institutions acted as facilitators, with necessary administrative approvals obtained beforehand.

Training and supervision

PG residents were initially sensitized to systematic reviews during journal club sessions. They then introduced the concept to the undergraduate (UG) students under faculty supervision. Faculty members provided three systematic review articles for the PG residents to present in journal clubs. Doubt-clearing sessions were conducted via Zoom, while WhatsApp, Google Groups, and email facilitated ongoing interactions.

Systematic review process

Each large group (five students) began by defining the objectives and inclusion/exclusion criteria for their systematic review. They independently searched for and downloaded relevant articles, which were then screened according to the predefined criteria. Faulty designs were excluded with the help of supervising faculty members. Students prepared Preferred Reporting Items for Systematic Reviews and Meta-Analyses (PRISMA) flowcharts and posted them in the WhatsApp group for facilitator feedback.

Article evaluation and consensus building

Students evaluated the downloaded articles based on a checklist covering various aspects such as title, study location, participant number, inclusion/exclusion criteria, sampling technique, study objectives, type of self-directed learning (SDL) used, study duration, data acquisition technique, evaluation tools, results, strengths, and limitations. Each group reached a consensus and posted their findings in the WhatsApp group for faculty review and further modification.

Feedback and program evaluation

At the module's conclusion, feedback was collected from the students on a self-administered questionnaire developed and face-validated by six experts. The questionnaire has 10 statements with agree and disagree closed-ended response options. The program was evaluated by another 15-item questionnaire formulated according to the Kirkpatrick model [11].

Data analysis

Data were presented in numbers. Percentages were not calculated as the sample was below 100. The categorical data were compared by a binomial test where significance indicates that the occurrence did not happen by chance. We used free online tools for statistical analysis according to the guidance by Mondal et al. [12]. A p-value <0.05 was considered statistically significant.

## Results

A total of 30 undergraduate medical students with a mean age of 22.7±0.95 years participated in the study. All six groups could formulate objectives, decide on inclusion and exclusion criteria, determine the search strategies, write methodologies for the search of the literature of the systematic reviews, prepare PRISMA flow charts, download articles from the databases, review articles, and analyze them according to the guidelines provided. Their feedback regarding the module is shown in Table 1.

**Table 1:** Feedback from the students regarding the online elective module on systematic review

Statement	Response		p-value
	Agree	Disagree	
The online module gave me a greater opportunity to communicate with other students as there were no geographical and time constraints.	29	1	<0.0001
The online module was more engaging than the offline sessions for review of the literature.	21	9	0.02
I am more comfortable with online activities for literature review than the offline sessions and I feel that it is more convenient.	27	3	<0.0001
More topics should be covered using this educational model.	28	2	<0.0001
The online elective module has increased my confidence in literature review and the knowledge that I have gained will be applied in research activities in the future.	25	5	0.0002
I am motivated to learn research activities in online mode.	23	7	0.0026
The online module has improved my learning of literature review.	22	8	<0.0001
The flipped classroom has improved my problem-solving skills and critical-thinking skills.	28	2	<0.0001
The online module has helped me to more effectively apply my knowledge of literature review.	26	4	<0.0001
I am satisfied with the overall learning experience.	30	0	-
Overall (sum of 10 attributes/10)	25.9	4.1	-

The evaluation of the online systematic review training module for medical students revealed a highly positive reception. Students appreciated the enhanced communication opportunities without geographical and time constraints and found the online sessions more engaging and convenient than traditional offline methods. They expressed a strong desire for more topics to be covered using this educational model and reported increased confidence and motivation in conducting literature reviews and research activities. The flipped classroom approach was particularly effective in improving their problem-solving and critical-thinking skills, leading to an overall high level of satisfaction with the learning experience.

The students also evaluated the program, and their evaluation of various aspects of the program is shown in Table 2.

**Table 2:** Program evaluation according to the Kirkpatrick model

Evaluation Category	Response		p-value
	Agree	Disagree	
The objectives were clearly defined for this program	29	1	<0.0001
The objectives were covered by facilitators	28	2	<0.0001
The materials were of the right level of complexity for my background	25	5	0.0002
I understood the learning objectives of the elective	30	0	-
I was able to relate the learning objectives to my learning experience	20	10	0.049
I faced appropriate challenges with the material	25	5	0.0002
The course materials of the elective module were well-organized	28	2	<0.0001
I found the module easy to navigate	23	7	0.0026
I felt that the lesson I learned would be essential for my success	29	1	<0.0001
The module was relevant to my needs	28	2	<0.0001
I will be immediately able to apply the knowledge that I have gained	2	28	<0.0001
The facilitators had a good understanding of the systematic review process	30	0	-
The facilitator shared his/ her experience regarding the conduction of a systematic review	30	0	-
My learning was enhanced by the knowledge and guidance of the facilitator	26	4	<0.0001
My learning was enhanced by the experiences on systematic review shared by the facilitator	25	5	0.0002
Overall (sum of 15 attributes/15)	37.8	7.2	-

The evaluation of the elective module suggests clarity and relevance of the program's objectives, which were well-covered by facilitators. The materials were generally deemed appropriate in complexity and well-organized, although some students found the module challenging to navigate. The facilitators' deep understanding and experience with systematic reviews greatly enhanced students' learning. While most students found the lessons essential for their success and immediately relevant, a significant number felt unprepared to apply the knowledge gained right away. Overall, the module was positively received, with facilitators' guidance playing a crucial role in the learning process.

## Discussion

The evaluation of the online systematic review training module for final-year medical students revealed positive feedback. Students appreciated the clear definition and coverage of objectives, the appropriate complexity of materials, and the relevance of the module to their educational needs. They particularly valued the facilitators' expertise and the organization of the course materials. However, while the module was well-received in terms of enhancing communication, engagement, and problem-solving skills, some students felt unprepared to immediately apply the knowledge gained from the module.

The positive reception of the online module can be attributed to several key factors. Maybe the flexibility of online learning allowed students to engage with the material without the constraints of location and time, which is particularly beneficial given their busy schedules and limited time for electives [13-15]. The well-structured content and the use of experienced facilitators helped create a supportive learning environment. Additionally, the use of interactive platforms such as Zoom and WhatsApp facilitated continuous communication and feedback, enhancing the overall learning experience [16,17].

A study conducted by Pathipati and Taleghani in 2016 revealed that medical students may not be interested in research during their undergraduate course. Hence, the authors suggested that stakeholders should enhance the reward for research [18]. In our study, the electives are part of the medical curriculum, and we trained them for a systematic review that requires fewer resources than original research work. Another study by Murdoch-Eaton et al. found that while undergraduate students recognize the importance of research experience, they need practical and in-depth understanding [19]. In our study, we found that the developed model to teach systematic review was well accepted by the students. Hence, the same can be helpful for undergraduate research training in other centers. The detailed module can be obtained from the first author of the article.

The implications of this study are substantial for medical education. The success of the online module suggests that similar approaches could be adopted for other elective courses, especially in contexts where time and resource constraints are significant. Moreover, the positive outcomes related to communication, engagement, and critical-thinking skills highlight the potential of online learning to complement traditional educational methods [20]. Future research should explore the long-term impact of such modules on students' professional development and their ability to apply learned skills in clinical practice.

This study has several limitations. The sample size was small, comprising only 30 students from a single MBBS batch, which may not be representative of the broader student population. The reliance on self-reported data introduces potential biases, and the short duration of 15 days may not have been sufficient for students to fully adapt to the online learning environment. Additionally, the absence of a control group limits the ability to compare the online module's effectiveness against traditional methods. Variability in students' access to technology and internet quality could have affected their engagement and performance. Finally, focusing on a single department and specific topics restricts the generalizability of the findings to other areas of medical education. When designing studies in the future, researchers should take into account these limitations and incorporate strategies to address them in order to obtain a more generalized result.

## Conclusions

This study demonstrates the potential of online systematic review training modules to effectively enhance the learning experience for final-year medical students, particularly under the constraints of limited time and resources. The module was well-received, with students appreciating the clear objectives, the appropriate complexity of materials, and the facilitators' expertise. The flexibility and convenience of online learning, combined with interactive and well-organized content, significantly improved communication, engagement, and critical-thinking skills. Despite the limitations, these findings suggest that online learning can complement traditional educational methods, offering a viable solution to logistical challenges in medical education. Future research should aim to address the identified limitations and explore the long-term impact and broader applicability of such online modules in various medical fields.
